# The Impact of Ramadan Fasting on Endothelial Function, Cardiovascular Risk Factors, and Cardiovascular Disease

**DOI:** 10.3390/jcm14176191

**Published:** 2025-09-02

**Authors:** Musaab Ahmed, Mohamed H. Ahmed, Nisha Shantakumari

**Affiliations:** 1 College of Medicine, Ajman University, Ajman P.O. Box 346, United Arab Emirates; n.kumari@ajman.ac.ae; 2 Department of Geriatric Medicine, Milton Keynes University Hospital NHS Foundation Trust, Eaglestone, Milton Keynes MK6 5LD, UK; mohamed.hassan-ahmed@mkuh.nhs.uk; 3 Department of Medicine and HIV Metabolic Clinic, Milton Keynes University Hospital NHS Foundation Trust, Eaglestone, Milton Keynes MK6 5LD, UK; 4 Honorary Senior Lecturer of the Faculty of Medicine and Health Sciences, University of Buckingham, Buckingham MK18 1EG, UK

**Keywords:** Ramadan fasting, endothelial dysfunction, heart failure, atherosclerosis

## Abstract

Endothelial dysfunction, marked by reduced nitric oxide bioavailability, is a known factor in cardiovascular disorders. Prior studies have shown that dietary changes affect endothelial function and alter risk factors. Research studies have examined the possible impacts of Ramadan fasting on endothelial dysfunction. This narrative review aimed to assess the impacts of Ramadan fasting on endothelial function. We conducted a search of the PubMed and SCOPUS databases with various search terms. The review focused on research papers published in English language from 2000 to 2024. This review included sixty-six studies. Ramadan fasting elevated the levels and bioavailability of nitric oxide and improved some indicators of endothelial dysfunction. Ramadan fasting may enhance endothelial dysfunction and modify its risk factors, by promoting weight reduction and improving insulin resistance. Moreover, Ramadan fasting induced alterations in the structure, composition, abundance, and overexpression of gut microbiota. Further research is necessary to evaluate the safety, efficacy, and importance of Ramadan fasting on endothelial function and cardiovascular health.

## 1. Introduction

The blood vessel endothelium is a layer of cells that lines the internal layer of vessels, preserving vascular homeostasis via several protective functions including modulation of vascular tone and permeability, as well as anti-thrombotic, antioxidant, anti-inflammatory, and anti-proliferative activities [1]. The endothelium stimulates the secretion of nitric oxide (NO) and prostacyclin and endothelium-derived hyperpolarization which collectively inhibit the migration and proliferation of smooth muscle cells, impede platelet adhesion and aggregation, and modulate biological processes associated with thrombogenesis [2]. Endothelial dysfunction is defined by a change in endothelial actions and includes several maladaptive alterations in the functional phenotype, leading to adjustments to the control of thrombosis, vascular tone, inflammation, and hemostasis [3].

All cardiometabolic risk factors, including dyslipidemia, abdominal obesity, hypertension, and insulin resistance, are linked with endothelial dysfunction [4]. Endothelial dysfunction leads to the progression of atherosclerosis and might exacerbate cardiometabolic diseases. In individuals with diabetes, accumulation of glucose in the endothelial cells contributes to the activation of mechanisms that result in inflammation, the production of reactive oxygen species (ROS), and impaired nitric oxide (NO) synthesis, all of which facilitate the progression of atherosclerosis, coronary artery disease, and ischemic cerebral events [5].

Endothelial function can be measured by Intracoronary Artery Infusions using acetylcholine or other vasoactive substances, flow-mediated vasodilation, or peripheral arterial tone [6].

Ramadan fasting offers several health advantages, including decreasing body weight and composition, as well as improvements in the elements of metabolic syndrome especially insulin resistance. Moreover, fasting during Ramadan improves blood glucose levels and reduces oxidative stress and inflammatory markers [7]. This narrative review aims to discuss the pathophysiology, biomarkers, and risk factors of endothelial dysfunction and the potential effects of fasting during Ramadan on endothelial dysfunction and related cardiovascular disorders and gut microbiota.

## 2. Methods

**Figure 1:**
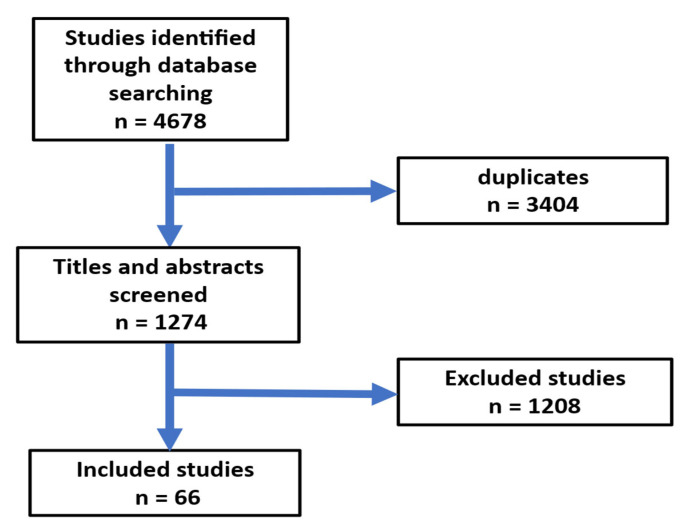
Flow chart for the selection of publications.

In this narrative review, the authors performed a search of the PubMed and SCOPUS databases using the following keywords: endothelial dysfunction, gut microbiota, atherosclerosis, blood pressure, heart failure, insulin resistance, obesity, diabetes, stroke, oxidative stress, and Ramadan fasting. The authors searched with a combination of the following terms: [Ramadan Fasting AND endothelial dysfunction OR Gut microbiota OR atherosclerosis OR heart failure OR body weight OR obesity OR diabetes OR glucose OR stroke OR blood pressure OR Oxidative stress AND clinical trials]. This review literature search focused on research publications published in English between 1 January 2000 and 30 August 2024. Article and abstracts were evaluated. Relevant articles were meticulously examined, and data concerning Ramadan intermittent fasting, study duration, participant count, and effects on endothelial dysfunction, gut microbiota, atherosclerosis, obesity, diabetes, atherosclerosis, blood pressure, heart failure, and oxidative stress were extracted. The reference list included pertinent citations. The original search produced a total of 4678 articles. A total of 66 papers were chosen for this review (Figure 1).

The following selection criteria were used for the selection of the articles.

Inclusion criteria: Observational studies, randomized controlled trials, experimental research, and review papers were included into the review. Only publications authored in English were eligible. All research concerning endothelial dysfunction, gut microbiota, heart failure, atherosclerosis, blood pressure, obesity, diabetes, stroke, oxidative stress, and Ramadan fasting was considered.

Exclusion criteria: Letters to the editor, comments, news articles, case reports, books, notes, theses, brief surveys, duplicate studies, conference abstracts, and publications not in English were excluded.

All gathered documents were imported into EndNote for the removal of duplicates. Consequently, abstracts and titles were assessed, and article selection was performed according to the predetermined eligibility criteria.

## 3. Pathophysiology of Endothelial Dysfunction

The pathophysiology of endothelial dysfunction is complicated and encompasses multiple mechanisms.

Oxidative stress significantly contributes to the pathophysiology of endothelial dysfunction. It originates from many enzymatic pathways, such as uncoupled eNOS, xanthine oxidase, NADPH oxidases, and dysfunctional mitochondria, and takes place when the balance between antioxidant and prooxidant systems is disturbed [8]. Elevated levels of reactive oxygen species (ROS) oxidize intracellular macromolecules, subsequently decreasing nitric oxide (NO) production through the formation of peroxynitrite leading to the degradation of the eNOS cofactor tetrahydrobiopterin, causing the uncoupling of endothelial nitric oxide synthase (eNOS) and promoting prooxidant activity. The oxidative stress is associated with diminished endothelial vasodilation and a pro-inflammatory condition. Additionally, it increases the expression of adhesion molecules, such as ICAM-1 and VCAM-1, and chemotactic molecules [9]. Inflammation is a significant factor in the pathogenesis of cardiovascular disease [10]. Endothelial cells produce various molecules in response to injury, including interleukin-8, interferons, chemokines, colony-stimulating factors, monocyte chemoattractant protein-1, intercellular adhesion molecule-1, E-selectin, growth factors, P-selectin, vascular adhesion molecule-1, and other inflammatory factors [8]. This results in the enhanced adhesion and migration of leukocytes through the endothelium, leading to activation of a pro-inflammatory state [11,12]. Additionally, pro-inflammatory mediators induce endothelial cells to release further pro-inflammatory cytokines, thereby perpetuating a vicious cycle [13]. This modification leads to a prothrombotic, proliferative, and pro-inflammatory condition typical of dysfunctional endothelial cells.

Metabolites of the gut microbiota have been demonstrated to exhibit varying effects on endothelial function. Specifically, intestinal bacteria can influence the endothelium of the vascular system via two primary mechanisms: firstly, the microbiota and its metabolites may activate the enteric nervous system, thereby affecting the brain centers that regulate cardiovascular function; secondly, they can translocate into the bloodstream through the blood–intestinal barrier, thereby modulating the function of tissues and organs that maintain circulatory homeostasis. Consequently, maintaining the microbial composition in a condition of eubiosis and mitigating intestinal dysbiosis has been suggested as an approach to diminish vascular and endothelial dysfunction [14].

## 4. Biomarkers of Endothelial Dysfunction

Various markers for endothelial dysfunction have been examined to forecast cardiovascular diseases, detect microvascular and macrovascular damage, and assess the progression of atherosclerosis. Endothelial adhesion molecules, including vascular cell adhesion molecule-1 (VCAM-1), intercellular adhesion molecule-1 (ICAM-1), P-selectin, and E-selectin, are released in response to pro-inflammatory cytokines including interleukin (IL)-1β, IL-6, C-reactive protein, and TNF-α, thereby facilitating the trans-endothelial migration of leukocytes [15,16].

Additional possible markers include oxidized LDL (Ox-LDL), asymmetric dimethylarginine (ADMA), and endothelial cell-specific molecule-1 (endocan). Ox-LDL activates the CD40/CD40L signaling pathway, thereby initiating inflammation [17]. In patients with hypertension, elevated levels of ADMA correlate with increased VCAM-1 and are proposed as a risk factor for stroke, kidney disease, and coronary artery disease [18]. Another possible marker is Angiopoietin-like protein-2 (ANGPTL2) which is expressed in atherosclerotic plaques, facilitating vascular inflammation and monocyte/macrophage chemotaxis through the nuclear factor-kappa B (NF-κB) signaling pathway [19].

In an experimental model of obesity, there is an elevated expression of some markers, including p21, p53, p16, tissue factor, VCAM-1, and sodium glucose transporters SGLT1 and SGLT2, correlating with diminished expression of eNOS [20].

## 5. Risk Factors of Endothelial Dysfunction

The risk factors of endothelial dysfunction include hypertension, heart failure, diabetes, dyslipidemia, obesity, insulin resistance, and MASLD [1]. Smoking is linked to vascular endothelial dysfunction. Smoking is believed to compromise vascular endothelial function by reducing nitric oxide bioavailability and activating oxidative stress and inflammation [21]. Other disease associated with endothelial dysfunction include atherosclerosis, stroke, and inflammatory bowel disease [8,22]. Gut microbiota plays an important in the pathogenesis of inflammatory bowel disease [23]. Endothelial dysfunction in stroke results in oxidative stress, inflammation, elevated vascular tone, blood–brain barrier disruption, and further cerebral problems. Endothelial dysfunction is a significant characteristic of chronic illnesses including atherosclerosis and hypertension. Endothelial function is known to be compromised early in atherosclerosis development, prior to any morphological alterations, a process closely associated with stroke [22]. Insulin resistance is a critical element in the onset of metabolic changes and endothelial dysfunction. Hyperglycemia results from peripheral insulin resistance, characterized by diminished insulin actions on peripheral tissues, leading to hyperinsulinemia to preserve normal glucose levels [24]. Insulin resistance is associated with elevated levels of uric acid, C-III, apo B, prothrombotic factors including fibrinogen and heparin-binding epidermal growth factor (EGF)-like growth factor, plasminogen activator inhibitor 1, homocysteine, and pro-inflammatory cytokines which contribute to vascular remodeling and endothelial dysfunction [25].

Hyperglycemia may inflict more damage on endothelial cells than on other cell types. In optimal circumstances, glucose mostly enters the cell via the GLUT-1 transporter, promoting ATP synthesis mainly via the glycolytic route. The activity of GLUT-1 is modulated by extracellular glucose levels. In diabetic individuals, GLUT-1 activity is heightened, leading to an augmented glycolytic flux that produces raised intracellular glucose levels and increased amounts of advanced glycation end products (AGEs). Excessive production of AGEs correlates with increased endothelial cell permeability, inhibition of endothelial NO synthase (eNOS) activity, and exacerbated detrimental modifications to DNA and proteins, resulting in cellular damage [26].

Hyperglycemia swiftly induces proliferation of endothelial cells by stimulating various pro-inflammatory and growth factors, including hepatocyte growth factor and vascular endothelial growth factor and rapid endothelial cell dysfunction-promoting transcriptional alterations [27].

Hyperglycemia and insulin resistance and lead to the excessive activation of renin–angiotensin–aldosterone system (RAAS), which contributes to the proliferation of smooth muscle cells, cardiac and vascular fibrosis, and remodeling, resulting in arterial stiffness [28,29]. Conversely, in hypoxic or inflammatory conditions, endothelial cells increase glycolytic flux to maintain oxygen and nutrient delivery to damaged regions. This results in elevated intracellular glucose levels, which activate the diacylglycerol/protein kinase C (DAG/PKC) signaling pathway, thereby increasing the permeability of the endothelial cell layer and facilitating leukocyte adhesion in various tissues [30]. Under these conditions, the production of prostaglandins, specifically prostaglandin H2 and F2α (PGH2 and PGF2α), along with thromboxane A2 (TXA2), enhances NADPH oxidase activity and the activity of type 4 and type 5 phosphodiesterases (PDE4 and PDE5). This leads to heightened production of reactive oxygen species (ROS) and the degradation of cyclic guanosine monophosphate (cGMP) and cyclic adenosine monophosphate (cAMP). This results in a diminished hyperpolarization and elevated levels of intracellular free calcium, promoting vasoconstriction [31].

Endothelial dysfunction contributes to the pathogenesis of heart failure with preserved ejection fraction (HFpEF). Hypertension, dyslipidemia, diabetes, and metabolic syndrome are common risk factors and comorbidities associated with HFpEF [32].

## 6. Gut Microbiota and Diseases

### 6.1. Gut Microbiota and Cardiovascular Diseases

The gut microbiome exhibits a complicated connection with cardiovascular diseases. Dysbiosis of the gut microbiota has been correlated with several cardiovascular diseases, including atherosclerosis, hypertension, and heart failure. The gut bacteria metabolize particular food constituents to generate metabolites like bile acids (BAs), Trimethylamine N-oxide (TMAO), and short-chain fatty acids (SCFAs). The concentrations of these metabolites in the bloodstream correlate with the risk of cardiovascular disease and affect several physiological activities, including inflammatory responses, regulation of blood pressure, lipid metabolism, and endothelial function [33,34].

### 6.2. Gut Microbiota and Hypertension

An experimental study by Li et al. [35] demonstrated that transplantation of fecal microbiota from individuals with hypertension into germ-free mice resulted in elevated blood pressure, thus affirming the impact of gut microbiota on blood pressure. In the same direction, Santisteban et al. demonstrated that dysbiosis of the gut microbiota augmented sympathetic neural connections between the gastrointestinal tract and paraventricular nucleus of the hypothalamus. Transplantation of gut microbiota from normal healthy rats into hypertensive rats decreased sympathetic activation and inflammation in the paraventricular nucleus, resulting in lowered blood pressure indicating that gut microbial metabolism may affect sympathetic drive via gut–sympathetic neural connections or by inducing neuroinflammation, thereby influencing blood pressure [36]. Hsu et al. [37] demonstrated that administration of sodium butyrate to pregnant mice on a tryptophan-free diet shown that sodium butyrate could avert hypertension in the offspring by enhancing the expression of GPR109A and GPR41 in the kidneys and re-establishing the equilibrium of the renin–angiotensin system. Metabolites generated by the gut microbiota are crucial intermediaries in the host–microbiota interaction that can influence blood pressure; these metabolites may exert both immune-dependent and immune-independent effects [38]. A study by Cai et al. [39] indicated that hypertensive patients demonstrate markedly diminished gut microbiota diversity and richness relative to healthy controls, characterized by a significant elevation in the Firmicutes/Bacteroidetes ratio. At the genus level, hypertensive individuals exhibit a substantial decrease in the relative abundance of Faecalibacterium, alongside an increase in the relative abundance of Enterococcus and Streptococcus, implying a potential association between hypertension and gut dysbiosis [39].

### 6.3. Gut Microbiota and Atherosclerosis

Dysbiosis of gut microbiota is regarded as a significant factor in the etiology of atherosclerosis. A study contrasting patients with atherosclerotic cardiovascular disease against healthy controls demonstrated that patient with atherosclerosis showed a relative reduction in the abundance of Bacteroides and Prevotella, while exhibiting a relative increase in Streptococcus and Enterobacteriaceae compared to healthy individuals, and there was a rise in the prevalence of oral cavity-associated bacteria, including Solobacterium moorei and Lactobacillus salivarius [40,41]. TMAO is regarded as the initial potential direct connection between atherosclerotic cardiovascular disease and gut microbiota [42]. TMAO can enhance the expression of scavenger receptor A1 (SR-A1) and cluster of differentiation 36 (CD36) in the body, resulting in the conversion of macrophages into foam cells in the wall of arteries to expedite plaque formation [43]. Zhu et al. [44] demonstrated that TMAO is capable of boosting cardiovascular disease risk by augmenting platelet reactivity and facilitating thrombosis, with increased TMAO levels independently correlating with the likelihood of thrombotic events in individuals. Furthermore, Selden et al. demonstrated that TMAO enhanced the expression of inflammatory genes by activating the NF-κB and MAPK signaling pathways, along with the NLRP3 inflammasome, thus facilitating the progression of atherosclerosis [45].

### 6.4. Gut Microbiota and Heart Failure

Growing evidence indicates a substantial involvement of gut microbiota and its metabolites in the development and progression of heart failure. The “gut–heart hypothesis” posits that in heart failure, impaired intestinal blood circulation results in hypoxia, ischemia, edema, and congestion of the intestinal mucosa. These modifications disturb the balance of gut microbiota, compromise the intestinal barrier, and enhance accessibility, facilitating the entry of microbial metabolites and gut bacteria into the bloodstream. This induces the secretion of pro-inflammatory cytokines, exacerbating chronic systemic inflammation, which subsequently impairs myocardial contractility, facilitates myocardial necrosis and cell death, and accelerates heart failure progression [34]. A Mendelian randomization analysis identified six microbial taxa indicative of a causal association with heart failure, with the most significant taxonomic unit being the Bacteroides dorei species [46]. Cui et al. [47] indicates a decline in microbial diversity among heart failure patients, characterized by an increase in Ruminococcus gnavus and a decrease in Faecalibacterium prausnitzii in their gut microbiota relative to healthy controls.

### 6.5. Gut Microbiota and Obesity

Dysbiosis of the gut microbiota might facilitate obesity by pathways that include increased fat synthesis, impaired energy metabolism, dysregulated appetite regulation, and low-grade inflammation. These interrelated pathways create a causal relationship between changes in gut bacteria composition and the development of obesity. The elimination of gut microbiota in obese mice through antibiotic treatment blunted weight gain and leptin resistance [34]. Moreover, the gut microbiome is closely linked to obesity. In comparison to the controls, reduced bacterial diversity and an elevated Firmicutes/Bacteroidetes ratio are typically considered fundamental traits of obese individuals [48]. An experimental study established that a heightened presence of short-chain fatty acid-producing bacteria, including Akkermansia, Alistipes, Bacteroides, and Phascolarctobacterium, can markedly diminish excessive fat accumulation and obesity in mice subjected to a high-fat diet, while also modifying the expression of critical genes associated with lipid metabolism [49]. Short-chain fatty acids can directly activate the G protein-coupled receptors GPR41 and GPR43 to enhance metabolic disorders. GPR41, an energy detector situated in the sympathetic nervous system and gastrointestinal tract, is stimulated by short-chain fatty acids such as propionate and butyrate. Upon activation, GPR41 induces enteroendocrine cells to release the peptide YY which enhances satiety and aids in appetite regulation. GPR43 is predominantly present in white adipose tissue and might directly impede insulin signaling in adipocytes, therefore diminishing fat storage in adipose tissue and enhancing lipid and glucose metabolism in other tissues [50,51].

### 6.6. Gut Microbiota and Type 2 Diabetes Mellitus

A review by Inayat et al. [52] showed that alterations in the gut microbiome are linked to insulin resistance, systemic inflammation, and reduced microbial diversity in type 2 diabetes mellitus. Zhou et al. [53] showed that the disruption of energy balance due to gut microbial dysbiosis is crucial in the advancement of type 2 diabetes. Mandaliya et al. [54] demonstrated that short-chain fatty acids, the metabolic byproducts of the gut microbiota, modulate insulin secretion and enhance proliferation of pancreatic β-cells in type 2 diabetes. Baars et al. [55] reported that composition of the gut microbiota in individuals with type 2 diabetes (T2D) significantly differs from that of those with normal blood glucose levels and the prevalence of Clostridia is markedly reduced, whilst the prevalence of Sutterella and Streptococcus is notably elevated. Subsequent research has shown that genera such as Clostridium, Ruminococcus, and Desulfovibrio facilitate the onset of type 2 diabetes, whilst genera like Bifidobacterium, Bacteroides, and Akkermansia might impede its advancement [55].

## 7. Ramadan Fasting and Endothelial Dysfunction

Ramadan fasting is a Muslim religious practice, taking place for one month each lunar year [56]. Ramadan fasting is a unique variant of intermittent fasting wherein Muslims are prohibited from consuming food, beverages, smoking, or taking medication from dawn until nightfall throughout the month of Ramadan [57,58]. Fasting during Ramadan is obligatory for all physically able adult Muslims, but the length of the daily fast during Ramadan varies by country and changes each year (8 to 16 h) [58]. Most individuals practicing Ramadan partake in two meals daily: Suhoor, before dawn to commence the fast, and Iftar, enjoyed after sunset to finish the fasting [59,60]. This will necessitate the need for careful consideration for those individuals with type 2 diabetes and willing to observe the fasting in order to mitigate risks like dehydration, hyperglycemia, or hypoglycemia [58,61]. Ramadan fasting has several health effects, including weight loss, decreased inflammatory markers and oxidative stress, reductions in blood pressure, and improved blood glucose levels [7,62].

Four studies assessed the effects of Ramadan fasting on endothelial dysfunction. A study in Iran involving twenty-one patients with cardiovascular illnesses was the first study to assess impact of Ramadan fasting on endothelial dysfunction. The biochemical parameters in the blood samples of patients were assessed two days before and after Ramadan fasting. The study demonstrated that nitric oxide (NO) levels were considerably elevated in patients following Ramadan fasting (85.1 ± 11.54 vs. 75.8 ± 10.7 μmol/L) (*p* < 0.05). The post-Ramadan concentrations of asymmetric dimethylarginine (ADMA) considerably decreased (802.6 ± 60.9 vs. 837.6 ± 51.0 nmol/L) (*p* < 0.05). Furthermore, the concentrations of vascular endothelial growth factor (VEGF) exhibited an increase, whereas malondialdehyde (MDA) levels demonstrated a decrease during Ramadan fasting; however, these alterations were not significant (228.1 ± 27.1 vs. 222.7 ± 22.9 pg/mL and 3.2 ± 0.7 vs. 3.6 ± 1.1 μmol/L, respectively) [63]. Another study in Iran assessed the impact of Ramadan fasting on the molecular marker of endothelial dysfunction, intercellular adhesion molecule-1 (ICAM-1), in both individuals with and without diabetes who fasted the whole month of Ramadan. Blood samples were taken 4 weeks before Ramadan and 2 weeks after the end of Ramadan. The study demonstrated a significant decrease in ICAM-1 levels in both groups [64]. Another study in Turkey examined the influence of fasting during Ramadan on endothelial dysfunction in 64 hypertensive patients who fasted the whole month of Ramadan. Flow-mediated dilation and biochemical parameters were measured 3 days before and after Ramadan. The study revealed a significant enhancement in flow-mediated dilation post-Ramadan, potentially attributable to reduced cortisol and CRP levels following the fasting period [65]. Goser et al. conducted a retrospective study involving 67 patients with sluggish coronary flow to evaluate the impact of Ramadan intermittent fasting on endothelial dysfunction, utilizing the TIMI frame count before Ramadan and one to three months after Ramadan [66]. The findings of the study indicated that Ramadan fasting and associated lifestyle modifications could enhance endothelial function [66]. Fasting during Ramadan may alleviate endothelial dysfunction and boost nitric oxide bioavailability (Table 1). Further research studies are needed to evaluate the effect of fasting during Ramadan on endothelial dysfunction.

### 7.1. Ramadan Fasting and Hypertension

Eight studies demonstrated impacts of fasting during Ramadan on blood pressure. The LORANS study in the UK involving 85 participants showed that systolic blood pressure and diastolic blood pressure post-Ramadan fasting decreased by 7.29 mm Hg even after controlling for the potential confounders. The effect of Ramadan fasting on blood pressure was independent of changes in fat mass, body weight, and total body water [67]. An observation study in Turkey including 129 patients with hypertension reported that Ramadan fasting produced a significant reduction in systolic and diastolic blood pressures in hypertensive patients using diuretics. Although blood pressure decreases, diuretics are typically well tolerated and may be safe for well-controlled hypertensive individuals during Ramadan fasting [68]. A study by Farag et al. [69] in Iraq with 120 hypertension patients receiving atenolol 50 mg per day demonstrated that fasting during Ramadan resulted in a significant notable reduction in blood pressure. The work of Samad et al. [70] included 40 normotensive individuals. Blood pressure readings were obtained one week before the commencement of Ramadan and on the 7th, 14th, and 21st days of Ramadan, including pre- and post-Iftar measurements. The study demonstrated that fasting during Ramadan produced a significant decrease in systolic and diastolic blood pressures in normotensive individuals. Similarly, the work of Bener et al. [71] involving 1246 diabetic individuals demonstrated that Ramadan fasting resulted in a significant decrease in both systolic and diastolic blood pressure. A study conducted by Ouselati et al. [72] involving fifty-two individuals with type 2 diabetes mellitus revealed that Ramadan fasting resulted in no significant alterations in blood pressure. Conversely, the work of Nematy et al. [73] in Iran with eighty-two Muslim patients with cardiovascular risk factor and fasting for Ramadan revealed a substantial reduction in systolic blood pressure. Regarding the effects of Ramadan fasting on ambulatory blood pressure, two studies were conducted. A study by Norouzy et al. [74] in Saudi Arabia, including 18 patients, indicated no significant differences in systolic and diastolic blood pressures between patients with hypertension and normotensive patients during Ramadan and one-month post-Ramadan. A study by Zairi et al. [75] including sixty treated hypertension patients showed no significant alterations in diastolic or systolic blood pressure.

Suggested mechanisms for blood pressure decrease by intermittent fasting involve activation of the parasympathetic nervous system [76], modulation of gut microbiota, and decreased activity of the renin–angiotensin–aldosterone system. Vascular endothelium potassium channels are crucial for the dilatation of blood vessels by inducing relaxation of vascular smooth muscle [77,78]. Obesity modifies the expression, sensitivity, and functionality of vascular potassium channels, resulting in smooth muscle dysfunction [79]. Intermittent fasting can lead to weight loss, modify potassium channel activity, mitigate vascular dysfunction, and reduce blood pressure.

### 7.2. Ramadan Fasting and Atherosclerosis

Only three studies reported the impacts of fasting during Ramadan on atherosclerosis. A study in Iran involving 50 healthy individuals showed that Ramadan fasting produced significant reduction in the levels of apoprotein B and the LDL-C/HDL-C ratio (*p* < 0.05), whereas triglycerides LDL-C, HDL-C, total cholesterol, and fasting blood glucose remained unchanged throughout the month. Fibrinogen levels and factor VII activity were considerably reduced over the month of Ramadan (*p* < 0.05). Coagulation alterations regarded as risk factors for atherosclerosis are mitigated during Ramadan [80]. Another study in the USA involving 14 patients with metabolic syndrome demonstrated modification in the expression of genes linked to lipid metabolism and atherosclerosis pathways. The genes exhibiting differential expression in the lipid and atherosclerosis pathways include APOB (*p* = 0.008), CD36 (*p* = 0.040), CALM1, CALM2, CALM3 (*p* = 0.015), and HSPA8 (*p* = 0.047). Ramadan fasting exerts anti-atherosclerotic effects [81]. A study in Egypt involving 103 obese individual showed that the Ramadan fasting model may serve as a behavior modification program to regulate or avert atherogenicity due to its beneficial effects on lipid profiles, hematological parameters, and coagulation metrics [82]. High-quality research studies are needed to investigate the effect of Ramadan fasting on atherosclerosis.

### 7.3. Ramadan Fasting and Diabetes Mellitus

Ten studies reported the impacts of fasting during Ramadan on glycemia. Tahapary et al. [64] demonstrated that fasting during Ramadan produced a significant decrease in ICAM-1 levels, in individuals with diabetes mellitus. A study conducted in Saudi Arabia by Al Hayek et al. [83] included 93 individuals with diabetes to evaluate the glucometric changes in individuals with type 2 diabetes mellitus before, during, and after fasting for Ramadan showed that fasting during Ramadan may improve glucose levels in individuals with type 2 diabetes mellitus who are not receiving intensive insulin therapy, while exhibiting a relatively low occurrence of hypoglycemia. A study by Ouselati et al. [72] found that the Ramadan fasting produced a significant reduction in plasma insulin level and fat body mass and no significant alteration in fasting plasma glucose in fifty-two individuals with type 2 diabetes mellitus. The work of Bener et al. [71] involving 1246 individuals with diabetes showed that Ramadan intermittent fasting produced a significant improvement in blood glucose and HbA1c. A study by Aljahdali et al. [84] involving sixty-eight individuals with diabetes demonstrated that Ramadan fasting produced an increase in the levels of HDL-C and a reduction in anthropometric measurements and the plasma levels of inflammatory cytokines. Ben Ahmed et al. [85] reported that Ramadan fasting produced a significant reduction in insulin resistance index, fasting plasma glucose, and insulin level in eighty-four patients with coronary artery disease. A study by Mohammadzadeh et al. [86] including thirty healthy individuals reported that fasting during Ramadan did not produce a significant decrease in fasting plasma glucose levels. The work of Abdullah et al. [87] including twenty-one healthy subjects reported that Ramadan intermittent fasting led to a significant decrease in insulin resistance. A study by Prasetya et al. [88] demonstrated that the observance of fasting during Ramadan significantly improved insulin sensitivity and did not cause significant changes in the plasma level of glucose in healthy men. The work of Selen et al. [89] in Turkey included10 male volunteers fasting for Ramadan. The study showed that Ramadan fasting produced a significant reduction in the plasma levels of triglycerides and a significant increase in levels of HDL-C and decreased levels of the fatty acid binding protein [89].

The results of the studies show inconsistent support for an enhancement in fasting blood glucose levels most likely due to glucose level variations during the day [90,91]. The observed variances in the outcomes of these studies may be attributed to the differing cohorts examined, specifically, individuals who are obese compared to those who are not, suggesting a notable influence based on subgroup differences.

### 7.4. Ramadan Fasting and Obesity

Twelve studies reported the effects of fasting during Ramadan on body fat and body weight. A study conducted by Khalil et al. [92] in Italy including 34 individuals demonstrated that Ramadan intermittent fasting not traditional fasting resulted in reductions in subcutaneous fat and liver steatosis. Gur et al. [93] demonstrated that Ramadan intermittent fasting reduced the thickness of visceral fat in pregnant women. Rahman et al. [94] showed that Ramadan fasting can significantly increase HDL and reduce body weight among healthy adult males. Madkour et al. [95] reported that fasting during Ramadan is capable of improving unfavorable metabolic disorders and oxidative stress among obese patients who are diabetic. Sadiya et al. [96] demonstrated that Ramadan fasting reduced body weight and waist circumference in patients with MetS. Zouhal et al. [97] reported that fasting during Ramadan reduced body composition index in obese males. The authors concluded that Ramadan intermittent fasting is capable of combating obesity. A study by Madkour et al. [98] involving fifty-seven overweight and obese individuals showed that fasting during Ramadan significantly decrease fat mass, body weight, fat mass, and waist and hip circumference. The work of Abdullah et al. [87] including twenty-one healthy subjects showed that Ramadan intermittent fasting produced a significant improvement in waist circumference, insulin resistance, body mass index, body weight, and visceral and subcutaneous fat thickness. A study by Maaloul et al. [99] with twenty obese individuals found that Ramadan intermittent fasting with concurrent training is capable of producing a greater enhancement in fat percentage, body weight, and waist circumference and a greater reduction in LDL, triglycerides, and total cholesterol. The work of Celik et al. [100] in Turkey involving thirty-two healthy subjects reported that Ramadan intermittent fasting caused a significant reduction in body mass index. Fasting plasma glucose levels increased within the normal lipid profile and no effect was reported on lipids. A study by Ouselati et al. [72] including fifty-two individuals with type 2 diabetes mellitus reported that Ramadan fasting caused a significant improvement in body weight, fat body mass, body mass index, waist circumference, and plasma insulin levels. Ramadan fasting causes a reduction in body fat and body weight.

### 7.5. Ramadan Fasting and Heart Failure

A limited number of studies have evaluated the effects of Ramadan on heart failure. A study by Salam et al. [101] across seven Middle Eastern countries involved 4157 patients with acute heart failure and indicated that patients who were admitted during Ramadan (three hundred and seven patients) demonstrated a significantly reduced occurrence of signs and symptoms of volume overload compared to those admitted to hospital in other months. Similarly, atrial arrhythmias were markedly less prevalent and plasma cholesterol concentrations were substantially diminished during Ramadan. Moreover, hospitalization during Ramadan was not independently associated with heightened immediate or one-year death rates. A study by Abazid et al. [102] in Saudi Arabia involved two hundred and forty-nine patients and evaluated the influence of Ramadan fasting on the symptoms of chronic cardiac failure with reduced ejection fraction. The research indicated that Ramadan fasting is often considered safe for most people with chronic HFrEF. Non-compliance with medication and dietary guidelines is substantially associated with decompensated cardiac failure during Ramadan. Similarly, the work of Alam et al. [103] in Pakistan with nine hundred and thirty-eight patients indicated that Ramadan fasting alleviated symptoms and enhanced the quality of life for those with chronic heart failure and proposed that patients with heart failure be urged to observe fasting throughout Ramadan. A study by Al Suwaidi et al. [104] in Qatar involving more than twenty thousand patients with congestive heart failure over a 10-year period to examine the impact of Ramadan fasting on hospitalization rates for congestive heart failure revealed no substantial disparity in hospital admission for congestive heart failure during Ramadan fasting relative to non-fasting months. A further study conducted by Al Suwaidi et al. [105] in Qatar, Kuwait, the United Arab Emirates, and Bahrain involved four hundred and sixty-five patients and examined the impact of Ramadan intermittent fasting on individuals with cardiac illness, including congestive heart failure. The research indicated that the effect of Ramadan fasting on stable cardiac patients is minimal. Individuals with stable cardiac conditions are typically capable of fasting. A study by Chamsi-Pasha and Ahmed [106] in Saudi Arabia with eighty-six patients aiming to examine the impact of Ramadan fasting on individuals with cardiac illness, including congestive heart failure, determined that the effect of fasting during Ramadan on stable patients with heart conditions is minimal and most patients with stable cardiac conditions can fast during Ramadan without any detrimental consequences. Alaarag et al. [107] conducted a study including one hundred and fifty-eight patients, indicating that fasting during Ramadan can be safe for low-risk individuals with chronic cardiac failure and reduced ejection fraction when supervised by a healthcare professional. Patients with chronic cardiac failure with a lower ejection fraction, particularly those with a history of chronic renal disease or coronary revascularization, may encounter a higher incidence of deleterious effects during Ramadan intermittent fasting [107]. Further research is required to assess the effects of Ramadan fasting on cardiac failure and the safety and effectiveness of intermittent fasting during Ramadan for patients with heart failure.

### 7.6. Ramadan and Stroke

Only five studies evaluated the effects of Ramadan fasting on stroke. A study by Assy et al., 2022 [108], in Egypt including 220 patients with diabetes and cerebrovascular stroke showed that no significant difference was observed in the overall frequency of cerebrovascular strokes (ischemic and hemorrhagic) between diabetic patients admitted during Ramadan and those admitted in the preceding or subsequent months. During Ramadan, there was a numerical rise in the incidence of ischemic stroke compared to hemorrhagic stroke; however, this difference was not statistically significant. A study by Zimhony et al., 2018 [109], including 4727 patients of ischemic stroke demonstrated that the month of Ramadan, especially during the initial two weeks, constitutes a distinct and ethnicity-specific risk factor for ischemic stroke hospitalizations among the fasting Bedouin Arab community. The work of Saadatnia et al., 2009 [110], in Iran including 162 patients demonstrated that fasting elevates the incidence of cerebral venous and sinus thrombosis. Individuals in good health often do not have issues related to cerebral venous and sinus thrombosis during fasting; however, those predisposed, such as individuals with hypercoagulable conditions and women utilizing oral contraceptives, may face heightened risk. A study by El-Mitwalli et al., 2010 [111], in Egypt involving 507 patients demonstrated that a significant alteration in the circadian rhythm of stroke start has been observed, transitioning from the interval of 6:00 a.m. to 12:00 p.m. to the timeframe of 12:00 p.m. to 6:00 p.m. during the month of Ramadan. The work of Alotaibi et al., 2022 [112], in Saudi Arabia including 1058 people reported no significant difference, overall, between individuals with ischemic stroke during Ramadan and those in non-Ramadan months. Nonetheless, ischemic stroke patients exhibited elevated National Institute Stroke Scale (NIHSS) scores at discharge and an increased number of ICU hospitalizations during Ramadan [112]. Further studies are needed to evaluate the effect and safety of Ramadan fasting on patients with the two types of stroke.

### 7.7. Ramadan Fasting and Gut Microbiota

Nine studies demonstrated the effect of Ramadan fasting on gut microbiota. A study conducted by Ozkul et al., 2019 [113] included nine subjects. The study was conducted throughout Ramadan, including roughly 17 h of daily fasting from dawn to sunset during a 29-day period. Stool samples were taken the day before the start of Ramadan and the last day of Ramadan, and high-glucose or high-fat food consumption was excluded. The study demonstrated that Ramadan intermittent fasting results in an elevation of A. muciniphila and the B. fragilis group, both regarded as healthy constituents of gut microbiota. A study by Mohammadzadeh et al., 2021 in Iran included 30 subjects [86]. The intake of food was evaluated by a three-day record before and after Ramadan. The study findings indicated that serum butyrate levels significantly rose over the month of Ramadan from 0.23 ± 0.02 mM to 0.46 ± 0.03 mM (*p* < 0.05). The gut Bacteroides and Firmicutes exhibited increases of 21% and 13%, respectively, following Ramadan in comparison to pre-Ramadan levels (*p* < 0.05) [86]. The study of Ali et al. 2021 involving 34 healthy adult participants (18 Pakistani and 16 Chinese) showed that in the absence of geographic separation, Ramadan fasting results in notable differences in the structure, composition, and alpha and beta diversities of gut microbiota between Pakistani and Chinese individuals, primarily due to substantial dietary discrepancies [114]. Furthermore, intermittent caloric restriction linked to Ramadan fasting seemingly influenced beta diversity and the occurrence of certain signature taxa [114]. An experimental study by Su et al., 2022 demonstrated that Ramadan fasting altered the gut microbiota composition in BALB/c mice (*p* < 0.01) and notably induced the overexpression of butyrate-producing Lachnospiraceae and Ruminococcaceae (*p* < 0.01), mirroring the effects observed in human participants [115]. The work of Saglam et al., 2023 [116], in Turkey involving 12 healthy adults indicated that Firmicutes were more prevalent in the gut microbiota prior to fasting among participants; however, their abundance was dramatically reduced at the conclusion of Ramadan fasting (*p* < 0.05). Proteobacteria exhibited a considerably elevated abundance at the conclusion of the month of Ramadan (*p* < 0.05). Fasting correlated with a notable reduction in the levels of seven genera: Blautia, Coprococcus, Dorea, Faecalicatena, Fusicatenibacter, Lachnoclostridium, and Mediterraneibacter. In contrast, the populations of two bacterial genera, Escherichia and Shigella, increased after the conclusion of the fasting month. The dietary consumption study revealed a negative connection in three comparisons: Ihubacter and protein (rho = −0.54, *p* = 0.0068), Fusicatenibacter and vegetables (rho = −0.54, *p* = 0.0042), and Intestinibacter and nuts (rho = −0.54, *p* = 0.0065). The findings indicate that, despite a uniform fasting duration during Ramadan, the meal choices of individuals can influence gut microbiota [116]. The work of Su et al., 2021 [117], in China included 30 healthy volunteers. The fasting duration lasted for 30 days throughout Ramadan, with daily fasting occurring from sunrise to sunset, roughly 16 h. The study demonstrated that Ramadan fasting induces significant alterations in the gut microbiome. The increase in butyric acid-producing Lachnospiraceae induced by intermittent fasting offers a clear molecular explanation for the health benefits linked to this dietary practice. A study by Ozkul et al., 2020 [118], in Turkey including nine healthy individuals with stool samples taken the day before start of Ramadan and the last day of Ramadan demonstrated that Butyricicoccus, Bacteroides, Faecalibacterium, Roseburia, Allobaculum, Eubacterium, Dialister, and Erysipelotrichi were considerably enriched taxa following the conclusion of Ramadan fasting. Random forest analysis indicates that the bacterial species most impacted by Ramadan fasting was Butyricicoccus pullicaecorum. The authors concluded that Ramadan fasting induces compositional alterations in the gut microbiota [118]. A study by Selen et al., 2024 [89], in Turkey involving 10 healthy volunteers demonstrated significant enhancement in both the alpha and beta diversity of the gut microbiota subsequent to Ramadan fasting (*p* < 0.05). The Firmicutes phylum, Clostridia class, Clostridiales order, and Ruminococcaceae family showed statistically significant reductions, whereas the Bacteroidetes and Proteobacteria phyla, Bacteroidia, Alphaproteobacteria, and Erysipelotrichi classes, Bacteroidales, Erysipelotrichales, and Actinomycetales orders, Erysipelotrichaceae family, and Prevotella genus exhibited statistically significant increases (*p* < 0.05). Ramadan fasting alters the gut mirobiota and enhances blood lipid profiles and FABP4 levels [89]. The work of Jo et al., 2023 [119], in South Korea involving 20 Muslim individuals was conducted to assess the composition of the gut microbiomes of the participants over the 4-week Ramadan period and the following 8-week post-Ramadan interval. Fecal microbiota analysis was performed, and short-chain fatty acids (SCFAs) were evaluated using liquid chromatography–mass spectrometry. The study demonstrated a reduction in SCFA levels and beneficial bacteria during Ramadan, coupled with heightened microbial diversity post-Ramadan, which indicates that the daily dietary intake during Ramadan might be insufficient in nutrients to sustain a healthy gut microbiota [119]. Most of the studies have a small sample size. High-quality studies with a larger sample size are needed to evaluate the effect of Ramadan on the gut microbiota.

### 7.8. Ramadan Fasting and Autophagy

Recent research studies have elucidated the significance of autophagy in the vascular endothelium, indicating that it is a crucial process enabling endothelial cells to adapt to stresses and fluctuations in blood flow. Autophagy significantly influences various essential elements of endothelial cell biology and vascular function, such as permeability, secretion, and angiogenesis. Dysregulation of autophagy in endothelial cells has been linked to the pathophysiology of various vascular disorders including endothelial dysfunction [120]. A recent study in the United Arab Emirates including fifty-one participants with overweight and obesity indicated that fasting during Ramadan is associated with increased expressions of autophagy-related genes (LC3B, LAMP2, ATG4D, and ATG5) which may partially clarify its advantageous short-term metabolic and health-promoting effects on early aging-related indicators. Thus, Ramadan fasting might have a protective benefit against early signs of metabolic illnesses in patients with overweight or obesity [121].

### 7.9. Ramadan Fasting and Oxidative Stress

**Figure 2:**
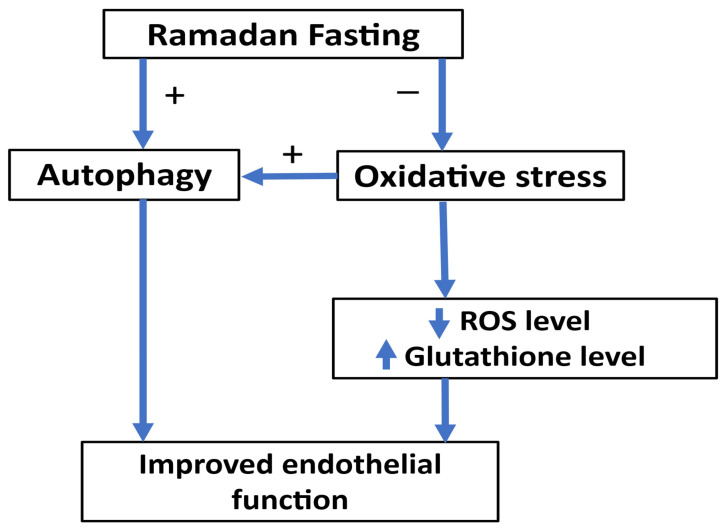
Relationship between Ramadan fasting, oxidative stress, autophagy, and endothelial function.

A study was conducted by Al-Shafei et al. [122] in Egypt involving 80 participants (40 diabetic and 40 non-diabetic). Fasting during Ramadan significantly increased blood glutathione levels in both groups. A study by Kasap et al. [123] in Turkey involving 100 healthy pregnant women (50 fasting and 50 are not fasting) reported that Ramadan fasting produced significant decrease in total antioxidant status in the fasting group and did not produce significant differences between the groups in total oxidant status and the oxidative stress index. The work of Faris et al. [124] in Jordan, involving 50 healthy participants, revealed that elevated body weight correlates with heightened lipid peroxidation and oxidative stress, with the influence of Ramadan intermittent fasting on oxidative stress being mediated by alterations in body weight. Another study by Al-Shafei et al. [125] in Egypt involving 40 hypertensive patients demonstrated that Ramadan fasting enhanced blood pressure and lipid profile, elevated glutathione levels, and mitigated oxidative stress in individuals with hypertension. The work of Ozturk et al. [126] in Turkey involving 72 pregnant women demonstrated that maternal fasting during the month of Ramadan in the second trimester does not significantly impact maternal oxidative stress. A study by Al Zunaidy et al. [127] in Saudi Arabia with 62 healthy women (31 premenopausal aged 21–42 years and 31 postmenopausal aged 43–68 years) revealed that Ramadan intermittent fasting correlated with enhanced markers of oxidative stress in both premenopausal and postmenopausal women. A study by Asemi et al. [128] in Iran involving 27 women with polycystic ovary syndrome demonstrated that Ramadan fasting produced significant increase in the plasma levels of NO and glutathione. The work of Mrad et al. [129] in Tunisia including 15 male patients with chronic obstructive pulmonary disease demonstrated that Ramadan fasting did not produce significant change in oxidative stress markers in patients with chronic obstructive pulmonary disease. A study by Asadi et al. [130] in Iran involving twenty-one patients with coronary artery disease, cerebrovascular, or peripheral arterial diseases demonstrated that Ramadan fasting produced a significant reduction in the plasma levels of serum amyloid A and protein carbonyl group. Another study in Turkey involving 57 healthy subjects demonstrated that Ramadan fasting significantly increased total antioxidant capacity and reduced total oxidant status and oxidative stress index [131]. Ramadan fasting is capable of reducing oxidative stress and might improve endothelial function (Figure 2).

## 8. Conclusions

**Figure 3:**
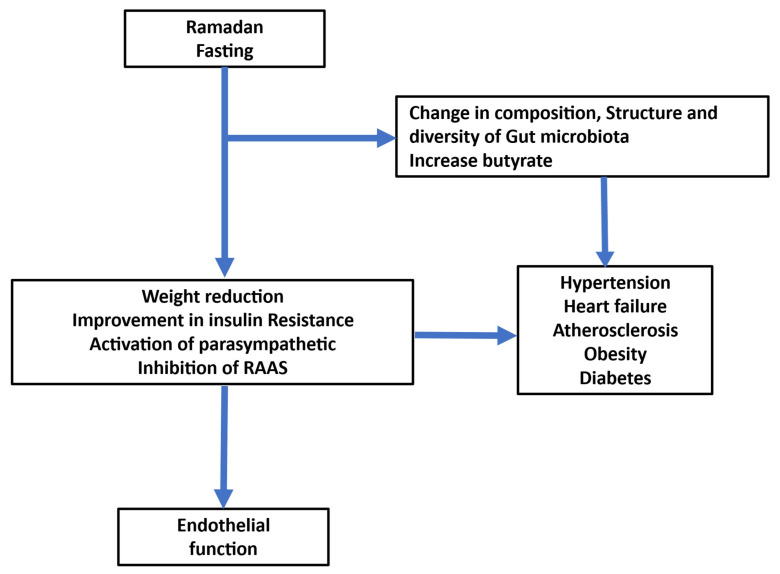
Relationship between Ramadan fasting, gut microbiota, and cardiac disorders related to endothelial dysfunction.

The pathophysiology of endothelial dysfunction is complex and involves different mechanisms. Cardiovascular risk factors and gut microbiota contribute to endothelial dysfunction. Ramadan fasting might improve endothelial function and increase the bioavailability of nitric oxide through weight reduction and improvement in insulin resistance (Figure 3). Moreover, Ramadan fasting might improve most of the risk factors related to endothelial dysfunction (Table 2). Furthermore, gut microbiota plays an important role in pathogenesis and the progression of risk factors of endothelial dysfunction. Ramadan fasting produces compositional alterations in the gut microbiota. Further studies are needed to assess and validate the effectiveness of fasting during Ramadan on endothelial dysfunction.

## Figures and Tables

**Table 1:** Summary of published studies on the impact of Ramadan fasting on endothelial dysfunction.

Authors	Main Finding	Reference
Demirci and Özkan 2023	Significant enhancement of flow-mediated dilation post-Ramadan is ascribed to reduced plasma levels of CRP and cortisol after Ramadan.	[65]
Tahapary et al., 2023	Fasting throughout Ramadan resulted in a decrease in ICAM-1 levels in both diabetic and non-diabetic individuals.	[64]
Goser et al., 2021	The study used the TIMI frame count to assess endothelial dysfunction and showed that Ramadan fasting improved endothelial dysfunction.	[66]
Yousefi et al., 2014	Levels of nitric oxide were significantly elevated in patients following Ramadan fasting. Post-Ramadan, asymmetric dimethylarginine (ADMA) levels significantly decreased, vascular endothelial growth factor (VEGF) levels elevated, and malondialdehyde (MDA) levels lowered during Ramadan fasting; however, these alterations were not statistically significant.	[63]

**Table 2:** Impacts of Ramadan fasting on cardiovascular risk factors of endothelial dysfunction and gut microbiota.

Impacts of Ramadan Fasting	
Cardiovascular Risk Factors by Decreasing the Following	Gut Microbiota
1. Blood pressure. 2. Apoprotein B and the LDL-C/HDL-C and fibrinogen and factor VIII. 3. Gene expression, lipid metabolism, and atherosclerosis pathways. 4. Insulin resistance. 5. Anthropometric measurements. 6. Plasma levels of inflammatory cytokines in individuals with diabetes. 7. Volume overload in heart failure patients. Atrial arrhythmias, and reduction in cholesterol serum levels improved the quality life and symptoms of chronic heart failure. 8. No significant difference in the overall frequency of cerebrovascular strokes between diabetic patients admitted during Ramadan and those admitted in the preceding or subsequent months. Rise in the incidence of ischemic stroke compared to hemorrhagic stroke during Ramadan. 9. Significant alteration in the circadian rhythm of stroke start has been observed, transitioning from the interval of 6:00 a.m. to noon to the timeframe of midday to 6:00 p.m. during the month of Ramadan.	1. Elevation of A. muciniphila and the B. fragilis group. 2. Serum butyrate levels significantly rose over the month of Ramadan, as well as gut Bacteroides and Firmicutes. 3. Significant differences in the composition, structure, and alpha and beta diversities of gut microbiota. 4. Overexpression of butyrate-producing Lachnospiraceae and Ruminococcaceae. 5. Reduction in the abundance of Firmicutes. 6. Proteobacteria exhibited a considerably elevated abundance. 7. Significant reduction in the levels of Blautia, Coprococcus, Dorea, Faecalicatena, Fusicatenibacter, Lachnoclostridium, and Mediterraneibacter. 8. Roseburia, Butyricicoccus, Bacteroides, Faecalibacterium, Dialister, Allobaculum, Eubacterium, and Erysipelotrichi were considerably enriched taxa following the conclusion of Ramadan fasting. 9. Reduction in SCFA levels.

## Data Availability

Not applicable.
